# Infantile Fibrosarcoma of the Upper Arm in a Neonate: A Rare Case Report

**DOI:** 10.7759/cureus.90827

**Published:** 2025-08-23

**Authors:** Prashant Bajaj, Sarita Dhankhar, Umesh Yadav, Chetan Agrawal, Ritesh Dhara, Shubham Verma, Nikhil Singh, Himanshu LNU, Ashish Devgun

**Affiliations:** 1 Orthopaedics, Pandit Bhagwat Dayal Sharma Post Graduate Institute of Medical Sciences, Rohtak, IND; 2 Orthopaedics, Vardhman Mahavir Medical College and Safdarjung Hospital, Delhi, IND; 3 Sports Medicine, Post Graduate Institute of Medical Education & Research, Rohtak, IND; 4 Orthopaedics, Post Graduate Institute of Medical Education & Research, Rohtak, IND

**Keywords:** infantile, infantile fibrosarcoma, paediatric orthopaedic, sarcoma, sarcoma soft tissue

## Abstract

Infantile fibrosarcoma (IFS) is a rare soft tissue malignancy typically presenting within the first year of life. It commonly affects the extremities but remains underreported in neonates. This case report aims to contribute to the limited literature on IFS involving the upper limb in neonates.

A one-month-old female presented with a gradually enlarging, painless swelling in the right upper arm since birth. Magnetic resonance imaging (MRI) suggested a soft tissue mass without bone involvement. Fine needle aspiration cytology (FNAC) resulted in a probable mesenchymal neoplasm. The mass was surgically excised and sent for histopathological evaluation. The final diagnosis revealed low-grade infantile fibrosarcoma (spinal muscular atrophy [SMA] negative, Ki-67 <2%). At one-year follow-up, the child had no recurrence or functional impairment.

Infantile fibrosarcoma, though histologically malignant, has a favorable prognosis with surgical management. Early recognition and complete excision are crucial for achieving excellent long-term outcomes. This case highlights the importance of considering IFS in the differential diagnosis of neonatal limb swellings.

## Introduction

Infantile fibrosarcoma (IFS) is a rare, low-grade soft tissue sarcoma of infancy, accounting for approximately 5-10% of pediatric soft tissue sarcomas [1]. The incidence of fibrosarcoma is estimated to be 0.3 per 100 000 population per year, and it accounts for less than 1% of all soft tissue sarcomas. It typically presents in children younger than one year of age, often within the first few months of life. Histologically, it may appear aggressive, yet the clinical behavior is relatively indolent, with a low metastatic potential compared to adult-type fibrosarcoma [2,3]. IFS predominantly affects the distal extremities and is usually congenital or manifests shortly after birth [1,4]. Despite this, upper arm involvement in neonates remains exceptionally uncommon, and clinical presentation may mimic benign tumors or vascular anomalies, leading to diagnostic delays [5,6]. Here, we present a rare case of IFS in a neonate's upper arm, successfully managed by wide surgical excision, with no recurrence at one year. This report adds to the limited data available and reinforces the importance of early intervention in such rare presentations.

## Case presentation

**Figure 1:**
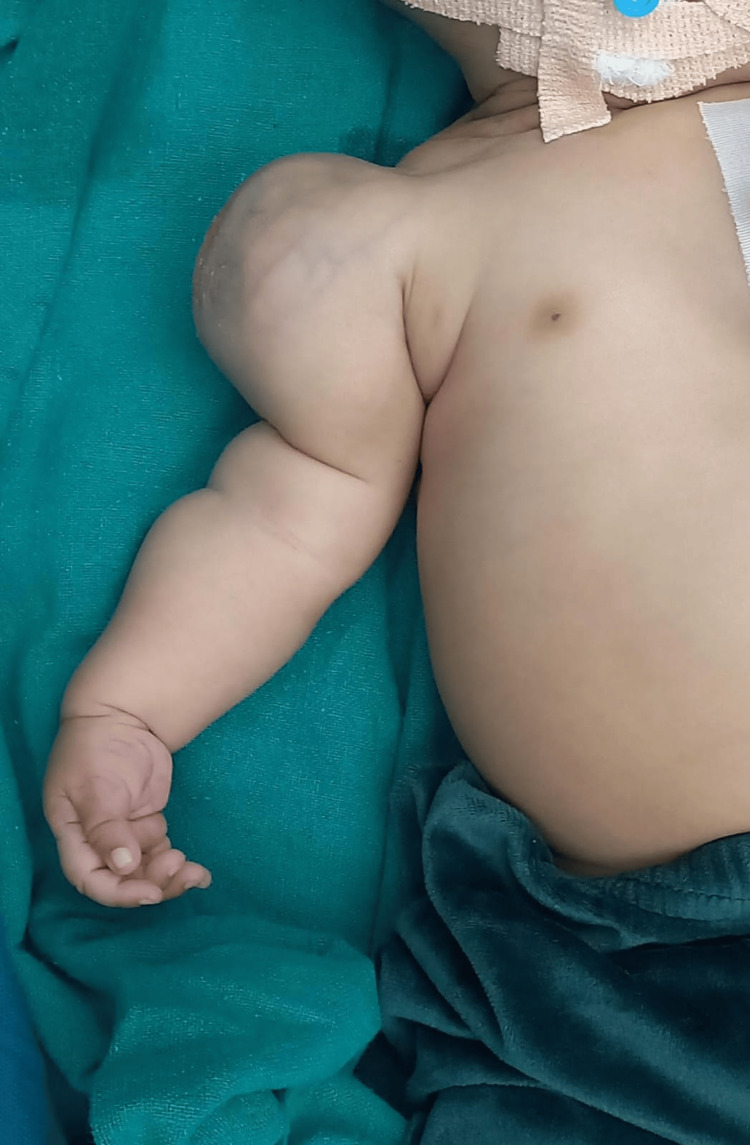
Soft tissue swelling over the right shoulder with dilated veins.

A one-month-old female infant was brought to our outpatient clinic with a painless, gradually increasing swelling over the right upper arm, noticed since birth (Figure 1).

**Figure 2:**
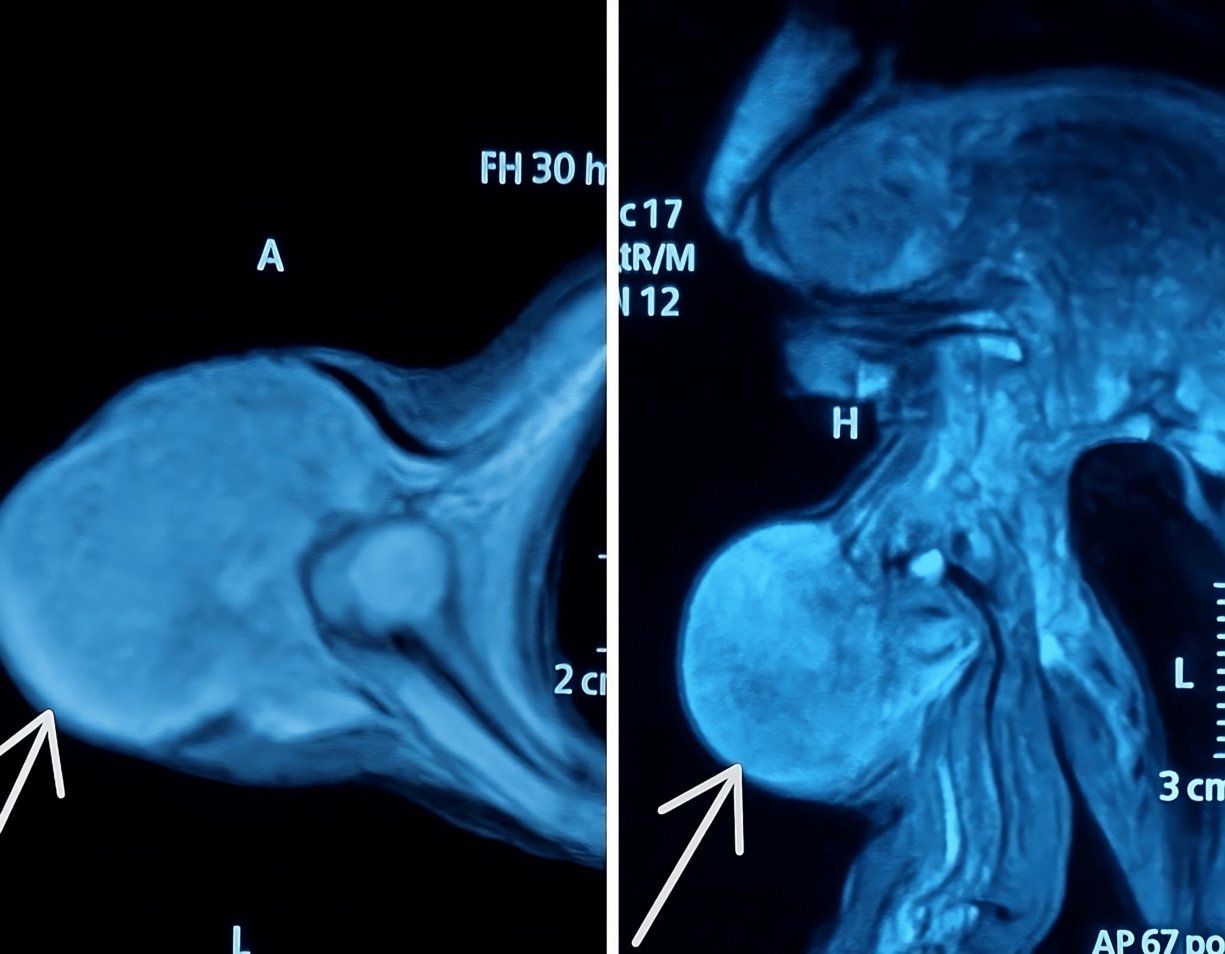
Axial and coronal section of MRI showing a large soft tissue mass (arrow) abutting the humerus bone.

There was no associated fever, trauma, or systemic symptoms. The baby was born at term via normal vaginal delivery with an uneventful perinatal period. Clinical examination revealed a firm, mobile, non-tender swelling over the anterolateral aspect of the upper arm, measuring approximately 4 × 3 cm with no palpable lymph nodes. The overlying skin was normal. Passive and active movements of the limb were intact, and no neurovascular compromise was noted. Magnetic resonance imaging (MRI) of the upper limb demonstrated a well-circumscribed, soft tissue lesion within the anterior compartment of the upper arm, isointense on T1 and hyperintense on T2, without adjacent bony erosion or neurovascular encasement (Figure 2).

**Figure 3:**
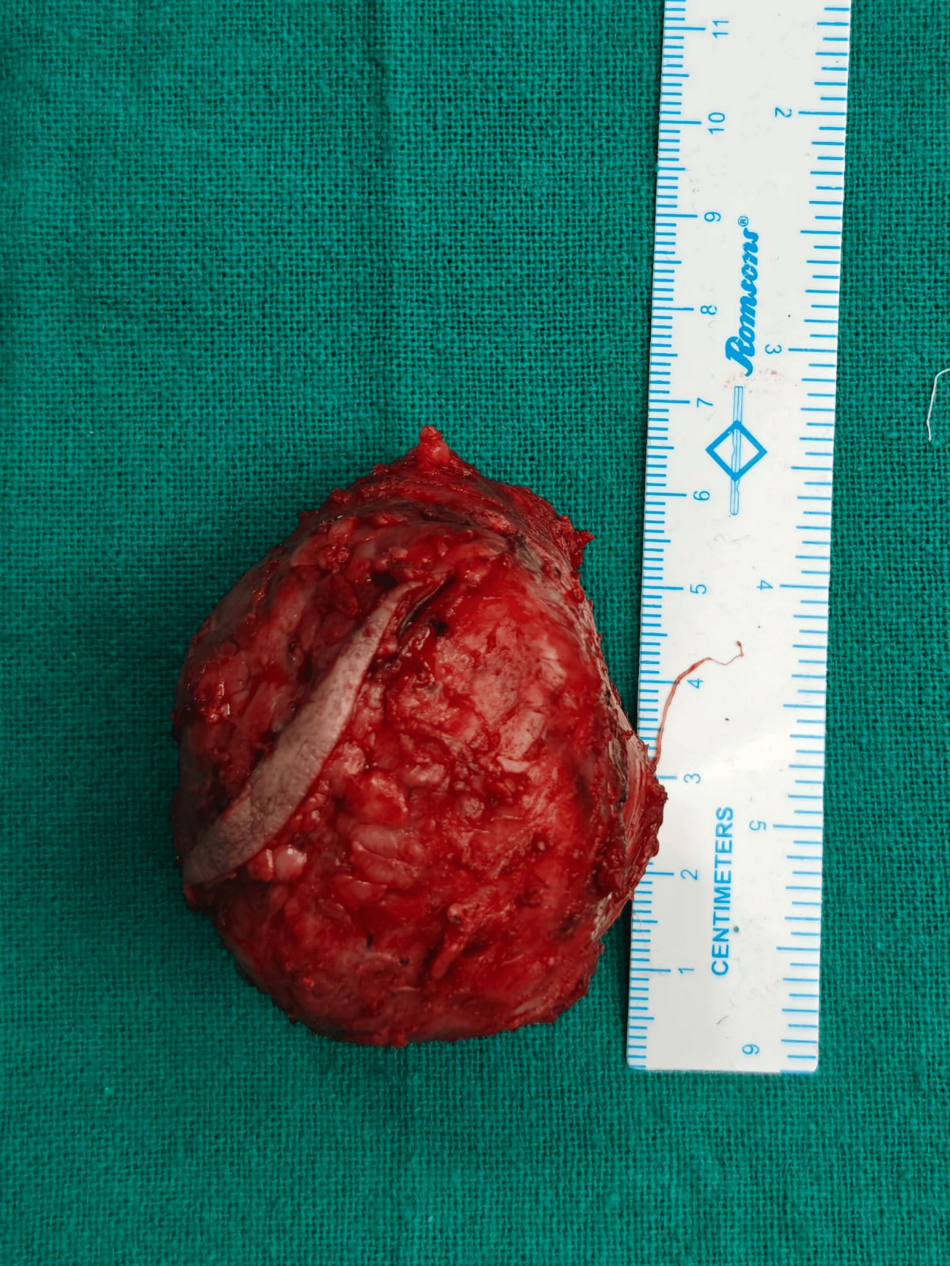
Excised tumor mass with small overlying skin.

The biopsy showed spindle-shaped mesenchymal cells suggestive of a neoplastic lesion, but was non-diagnostic. The patient underwent wide local excision of the mass under general anesthesia. Intraoperatively, the tumor was encapsulated and did not infiltrate surrounding muscles or neurovascular structures. The excised tissue of size 6.5 × 4 × 3.4 cm was sent for histopathological examination (Figure 3).

Histopathology revealed intersecting fascicles of spindle-shaped cells with mild nuclear atypia and low mitotic figures. Immunohistochemistry showed the tumor was negative for smooth muscle actin (SMA) and positive for vimentin, with a low Ki-67 index (<2%), confirming the diagnosis of low-grade infantile fibrosarcoma [3,7,8].

**Figure 4:**
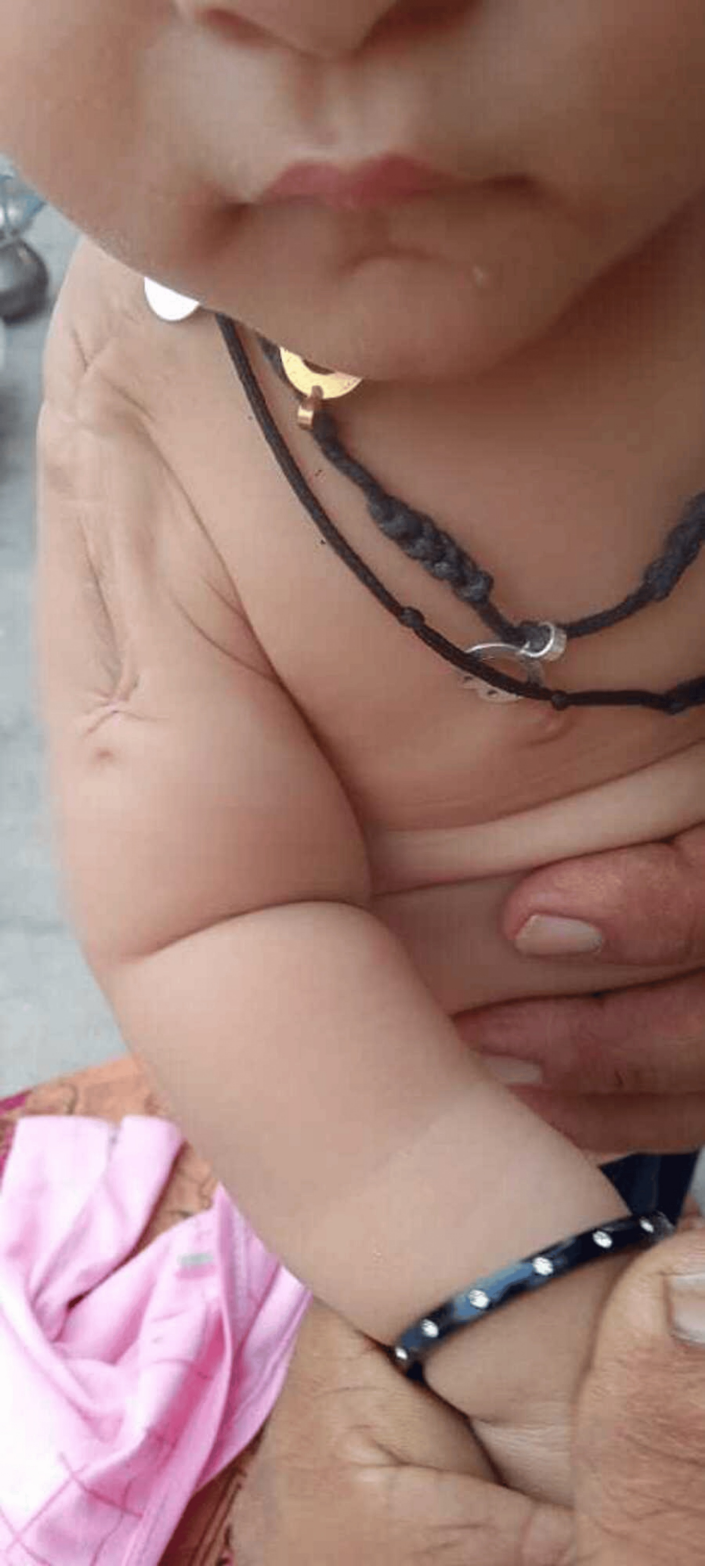
Follow up with normal skin and full functions.

The postoperative course was uneventful. At one-year follow-up, the patient remained disease-free with no clinical or radiological evidence of recurrence (Figure 4). Limb growth and function were preserved.

## Discussion

Infantile fibrosarcoma is a distinct pathological entity from adult fibrosarcoma and typically carries a better prognosis [2,3]. While most cases arise in the lower extremities, particularly the thigh or distal leg, involvement of the upper arm is extremely rare [5,9]. Congenital presentation, as in our case, is seen in approximately 40% of patients [1,6]. MRI is the imaging modality of choice and aids in surgical planning by delineating tumor margins and involvement of adjacent structures [4]. However, radiologic findings may not distinguish IFS from other soft tissue tumors. FNAC, or core needle biopsy, may yield suggestive features, but definitive diagnosis relies on histopathology and immunohistochemistry [6,10]. IFS typically exhibits spindle-shaped cells in fascicular arrangements and may show positivity for vimentin, desmin, and variable SMA [7]. Molecular genetics has emerged as a cornerstone in the contemporary management of sarcomas, with the identification of oncogenic gene fusions such as ETV6-NTRK3 guiding both diagnosis and therapy. The availability of highly selective TRK inhibitors (larotrectinib, entrectinib) has demonstrated durable responses across tumor types [3,8]. However, molecular testing was not available in our setting. The mainstay of treatment is complete surgical excision with negative margins [1,2]. Chemotherapy is reserved for unresectable, metastatic, or recurrent cases, and agents such as vincristine, actinomycin D, and cyclophosphamide have shown efficacy [3,4]. In our case, complete excision was achieved, and no adjuvant therapy was required.

Long-term follow-up is essential due to the risk of local recurrence, especially if margins are involved [2,3]. Our patient had no recurrence at one year, consistent with the literature indicating excellent outcomes for completely excised, low-grade tumors.

## Conclusions

IFS of the upper arm in neonates is an exceedingly rare clinical entity. Prompt diagnosis and wide local excision can lead to excellent long-term outcomes. Our case underscores the importance of including IFS in the differential diagnosis of congenital limb swellings and highlights the role of early surgical management in achieving disease control and functional preservation.
